# Aptamer based lateral flow biosensor for rapid detection of largemouth bass virus

**DOI:** 10.3389/fmicb.2025.1643764

**Published:** 2025-10-01

**Authors:** Xinyue Zhang, Lingfeng Guan, Zhenghong Xu, Hanchuan Wang, Xinyan Wei, Min Yang, Qiwei Qin, Shaowen Wang

**Affiliations:** ^1^College of Marine Sciences, South China Agricultural University, Guangzhou, China; ^2^Nansha-South China Agricultural University Fishery Research Institute, Guangzhou, China

**Keywords:** largemouth bass, LMBV, aptamer, lateral flow biosensor, rapid detection

## Abstract

The disease caused by Largemouth bass virus (LMBV) is one of the most serious viral diseases that threaten the development of largemouth bass industry. Point-of-Care testing of LMBV infection is crucial to prevent and control disease. Here, we designed a simple and sensitive aptamer-based lateral flow biosensor (LFB) method for the rapid detection of LMBV. In this method, two different aptamers (LA38s and LA13s) specific for LMBV particles were used for target capture and initiation of isothermal strand displacement amplification (SDA), respectively. After adding the reaction product to LFB, the color change of the control zone (C-line) and the test zone (T-line) can be observed by naked eyes within 5 min. The LFB possesses high specificity and accuracy for LMBV detection *in vivo* and *in vitro*. The detection limit of LFB is as low as 8 × 10^1^/mL LMBV infected cells, with the detection time of 1 h. Thus, this novel LFB provides a new detection tool, which can be used for rapid detection of LMBV in field.

## 1 Introduction

Largemouth bass (*Micropterus salmoides*) is one of the important economic fish species in China, and the production has reached 888,030 metric tons in 2023. However, with the increase of breeding density and the expansion of breeding scale, the outbreak of viral diseases is becoming more and more frequent.

Iridovirus is one of the large nucleoplasmic DNA viruses, which not only brings huge economic losses to the aquaculture industry, but also poses a serious threat to biodiversity (Whittington et al., 2010). Largemouth bass virus (LMBV) was initially isolated from diseased largemouth bass and characterized as genus *Ranavirus*, family *Iridoviridae* (Zhang et al., 2022a). It is the main viral pathogen seriously threatening the largemouth bass culture and causing huge economic losses. In addition, LMBV not only infects fish such as bluegill sunfish, red-breasted sunfish, striped bass, spotted bass, smallmouth bass and largemouth bass, but also amphibians such as salamanders and bullfrogs (George et al., 2015; Piaskoski et al., 1999). Largemouth bass infected with LMBV often showed different symptoms, include lethargy, abnormal movement, surface ulcers, hemorrhage, and spleen enlargement (Deng et al., 2011; Zhao et al., 2020; Liu et al., 2023). Hence, it is difficult to diagnose by symptoms of disease caused by LMBV.

Early diagnosis is crucial for virus control and prevention. Presently, several detection methods of LMBV have been developed, mainly including polymerase chain reaction (PCR), real-time quantitative PCR (qRT-PCR), loop-mediated isothermal amplification (LAMP) and isothermal recombinase polymerase amplification assay (RPA), as well as CRISPR/Cas based methods (Zhang et al., 2022b; Zhu et al., 2020, 2022; Guang et al., 2024; Li et al., 2025). Thereinto, conventional PCR and qRT-PCR are the most commonly used. Generally, these detection methods possess high sensitivity and reliability, but require special equipment, well-trained operator and complicated operation in the laboratory (Luo et al., 2014). Thus, an accurate and rapid method for LMBV on-site detection is still needed to ensure the healthy aquaculture of largemouth bass.

Point-of-Care testing (POCT) refers to a rapid and easy-to-use test occurring close the patient rather than in traditional laboratory, which is an attractive pattern for the on-site monitoring pathogen (Ferreira et al., 2018). Lateral flow biosensors (LFBs), also known as test strips, are a representative method in the POCT analysis, due to the noteworthy advantages of rapid, low-cost, user-friendly, high sensitivity and easy interpretation of results (Udhani et al., 2025). LFBs have been widely applied in multiple areas such as disease diagnosis, food safety and environmental assessments (Jauset-Rubio et al., 2017; Majdinasab et al., 2019; Xiao et al., 2021; Wang et al., 2024). Traditionally, most LFBs were designed based on antibodies. In recent years, aptamers have attracted more and more attention and have gradually become a new choice for bio-recognition element.

Aptamers are single-stranded oligonucleotide sequences (DNA or RNA) that can recognize various targets such as proteins, viruses, and bacteria, toxin and heavy metal ions (Zou et al., 2019; Santarpia and Carnes, 2024). In addition, aptamers also have the advantages of high sensitivity and specificity, easy modification, low cost, strong stability and short synthesis cycle, making them suitable as precision tools for diagnosing and treating diseases (Sujith et al., 2024; Majdinasab et al., 2022). Based on aptamers, a variety of LFBs have been developed for detecting various pathogens, such as *Escherichia coli*, *Salmonella*, Singapore grouper iridovirus (SGIV), red-spotted grouper nervous necrosis virus (RGNNV), and *Mycoplasma hyopneumoniae* (Fang et al., 2014; Liu et al., 2020, 2021; Ren et al., 2021; Zhang et al., 2022c).

In previous study, three DNA aptamers targeting LMBV particles were selected, and showed high sensitivity and specificity (Zhang et al., 2022a). From this, we further developed an aptamer based LFB combined with strand displacement amplification (SDA) and gold nanoparticle (AuNPs), for rapid detection of LMBV. This LFB can effectively detect different samples including LMBV particles, LMBV-infected cells, and LMBV-infected tissues. The detection limit reach as low as 8 × 10^1^ cells/mL, and the detection process can be completed within 1 h. Therefore, this novel LFB is a promising and effective detection method for on-site detection of LMBV.

## 2 Materials and methods

### 2.1 Reagents and materials

The bovine serum albumin (BSA) (A1933), trisodium citrate (S1804), HAuCl_4_ (254169), sucrose (S5016), Tween-20 (P9416), and Triton X-100 (93443) were purchased from Sigma-Aldrich (St. Louis, MO, USA). The streptavidin (SA) magnetic beads (Dynabeads™ MyOne™ Streptavidin C1)(65001) were purchased from Invitrogen (Carlsbad, CA, USA). The Nt.*Bbv*CI (R0632L) and the Klenow Fragment exo-DNA polymerase (M0212L) were purchased from New England Biolabs (Ipswich, MA, USA). The LA Taq^®^ (RR02MA) and deoxynucleoside triphosphates (dNTPs)(639125) were purchased from Takara (Shiga, Japan). The nitrocellulose membranes (SHF1350425) were purchased from Millipore (Burlington, MA, USA). The glass fiber (FH8975) and absorbent pads (FH-DB-8025-2) were purchased from Allway Biotech (Guangzhou, China).

### 2.2 Cells, virus, and aptamers

The fathead minnow (FHM) cells, grouper spleen (GS) cells and grouper brain (GB) cells used in this experiment were cultured in Leibovitz’s L15 (Gibco) medium containing 10% FBS and maintained in 28 °C (Huang et al., 2009, 2011).

LMBV isolated from diseased largemouth bass, was propagated in FHM cells and stored at −80 °C in our laboratory (Huang et al., 2014). Both SGIV and RGNNV were isolated from diseased grouper, propagated in GS and GB cells, respectively, and stored at −80 °C (Qin et al., 2001, 2003).

LA38 and LA13 are two specific aptamers targeting to LMBV particles. Through ELASA experiments, their Kd values were calculated to be 5.09 nM and 5.43 nM, respectively (Zhang et al., 2022a). To enhance the performance of these aptamers, the fixed primer sequences at both ends were truncated, and the truncated aptamers were named LA38s and LA13s, respectively. Compared to the original aptamers, the truncated aptamers demonstrated higher binding affinity to LMBV virions, with Kd values of 3.42 nM and 2.34 nM, respectively (Zhang et al., 2022b). In this study, LA38s were modified with biotin (Bio) and used as capture-aptamer. As an amplification aptamer, two specific sequences were added to LA13s for the SDA reaction. One sequence serves as the target site for the Nt.*Bbv*CI (*GCTGAGG*), while the other sequence acts as the binding site for the primers used in the SDA reaction (**TGGACACGGTGGCTTAGT**). All aptamers, probes and primer were listed in Table 1, and synthesized by Sangon Biotech.

**TABLE 1:** Oligonucleotide sequences used in this study.

Oligonucleotide	Sequence(5′-3′)	Bases (bp)
LA38s	GCCGGCCCGGGGGATAGAGTGCTCCCGATCCCTTGGCGAAGGGAC	45
LA13s	TTTTGACGCTTTATCCTTTTCTTATGGCGGGATAGTTTCG	40
Capture-aptamer	GCCGGCCCGGGGGATAGAGTGCTCCCGATCCCTTGGCGAAGGGAC-Bio	45
Amplification-aptamer	TTTTGACGCTTTATCCTTTTCTTATGGCGGGATAGTTTCG*GCTGAGG***TGGACACGGTGGCTTAGT**	65
AuNPs probe	SH-TATGGCGGGATAGTTTCG	18
C-line probe	CGAAACTATCCCGCCATA	18
T-line probe	TTTTGACGCTTTATCCTTT	19
SDA-primer	ACTAAGCCACCGTGTCCA	18

*GCTGAGG* represents the recognition site for Nt.*Bbv*CI enzyme, and **TGGACACGGTGGCTTAGT** is used for binding with SDA primers.

### 2.3 Preparation of AuNPs and AuNPs-probe conjugates

The synthesis procedure of AuNPs was carried out as previously described, with some modifications (Fang et al., 2014; Liu et al., 2021). Firstly, 200 mL HAuCl_4_ (0.01%) was added into a round bottom flask and placed on a magnetic stirrer for heating and stirring. Once the solution boiled, 4 mL of trisodium citrate (1%) was immediately added and stirred, and then the solution was continued to be heated and stirred until it turned wine red. Then, the solution was boiled for 5 min and cooled to room temperature with stirring. AuNPs were obtained, collected and stored in 4 °C with dark environment.

To prepare AuNPs-probe conjugates, AuNPs needs to be condensed. Briefly, 6 mL of AuNPs was centrifuged at 12,000 × *g* for 20 min, and the precipitate was collected by 900 μL PBS. Then, the thiol modified DNA (AuNPs probe, 10 μM, 100 μL) was added and gently shaken at 4 °C for 12 h. The solution was blocked by 10% BSA for 3 h, followed by the addition of 1.5 M NaCl and 1% sodium dodecyl sulfate (final concentrations of 150 mM and 0.01%, respectively). The solution was gently shaken at 4 °C for 12 h, and centrifuged at 12,000 × *g* for 20 min at 4 °C. The precipitate was gently resuspended in 1 mL of rinsing buffer (20 mM Na_3_PO_4_, 5% BSA, 0.25% Tween-20 and 10% sucrose). Finally, the solution was repeatedly centrifuged twice, and the precipitate was resuspended in 100 μL of rinsing buffer. The AuNP-probe solution was stored at 4 °C until use.

### 2.4 Construction of the LFB

**FIGURE 1:**
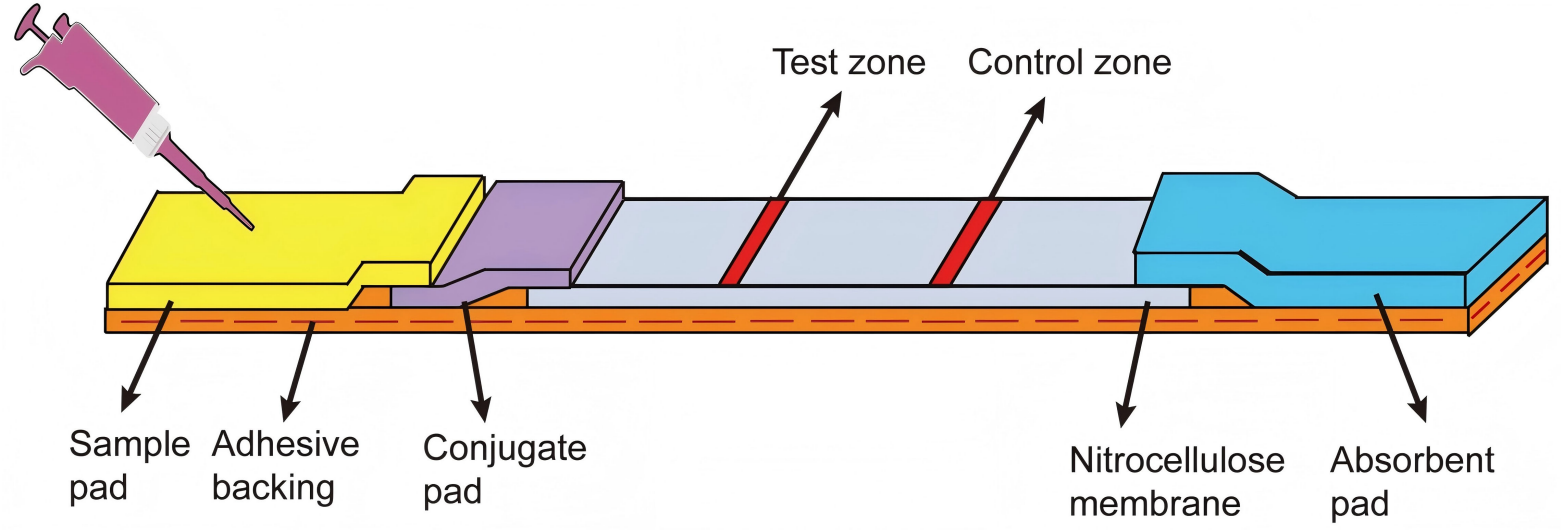
Configuration of the LFB. The sample pad, AuNPs-probe conjugate pad, nitrocellulose membrane and absorbent pad were assembled on the adhesive backing. The test zone (T-line) and control zone (C-line) were marked on the nitrocellulose membrane.

As shown in Figure 1, the LFB consisted of an adhesive backing, a sample pad, an AuNPs-probe conjugate pad, a nitrocellulose membrane, and an absorbent pad. Firstly, the sample pad was immersed in the sample pad buffer (0.5% Triton, 2% sucrose, 1% BSA, 50 mM boric acid, pH 8.0) for 2 h, then dried overnight at 37 °C for 12 h and stored at room temperature. Subsequently, the sample pad, conjugate pad, nitrocellulose membrane, and absorbent pad were adhered to the adhesive backing in turn, with an overlap of approximately 1.5 mm between adjacent parts. The T-line (T-line probe, 100 μM, 40 μL) and C-line (C-line probe, 100 μM, 40 μL) were dispensed onto the nitrocellulose membrane using the three-dimensional reciprocating gold sprayer (HM3260; Shanghai Kinbio, China) at a dispensing rate of 0.7 mL/cm, creating the C-line and T-line, respectively. The distance between the two probes was approximately 7 mm. Finally, the strip was cut into a size of 6 cm in length and 3.5 mm in width by a guillotine cutting machine (ZQ2002; Shanghai Kinbio, China) and stored at room temperature with a dry environment.

### 2.5 Virus-infected cell samples

To obtain cell samples for testing, cells (FHM cells, GS cells and GB cells) were cultured in a 24 well plates for 18 h. Then the virus (LMBV, SGIV and RGNNV) at a multiplicity of infection (MOI) of 0.5 was added, and the cytopathic effect was observed by optical microscope at 96 h post-infection. The virus-infected cell suspension was collected, centrifuged at 900 × *g* for 10 min, and lysed in Pierce IP™ Lysis buffer (Thermo Fisher Scientific, United States). Then they were washed with phosphate buffered saline (PBS; 137 mM NaCl, 2.7 mM KCl, 10 mM Na_2_HPO_4_, 1.8 mM KH_2_PO_4_, pH 7.4) and resuspended in 1 mL PBS. Uninfected cells were used as the control group. The cell samples were stored at −80 °C until use.

### 2.6 Virus-infected tissue samples

Largemouth bass (8–10 cm) were purchased from a farm in Foshan, Guangdong Province, China, and used for LMBV challenge experiments. They were maintained in a circulating water system at 28 ± 1 °C and fed twice daily. Before the challenge experiment, fish were randomly selected for virus detection to ensure that they did not infect LMBV. As previously described, largemouth bass were infected with 100 μL LMBV (10^4^.^7^TCID_50_/mL) by intraperitoneal injection. At 4 days post-infection, liver, spleen and kidney tissues were collected from diseased fish. After washing with PBS, the tissues were cut into small pieces and lysed in 300 μL of Pierce IP™ Lysis buffer (Thermo Fisher Scientific, United States) at 4 °C for 10 min. Then, the lysates were filtered through medical gauze, and the supernatants were collected for LFB detection.

### 2.7 Procedure for LMBV detection

**FIGURE 2:**
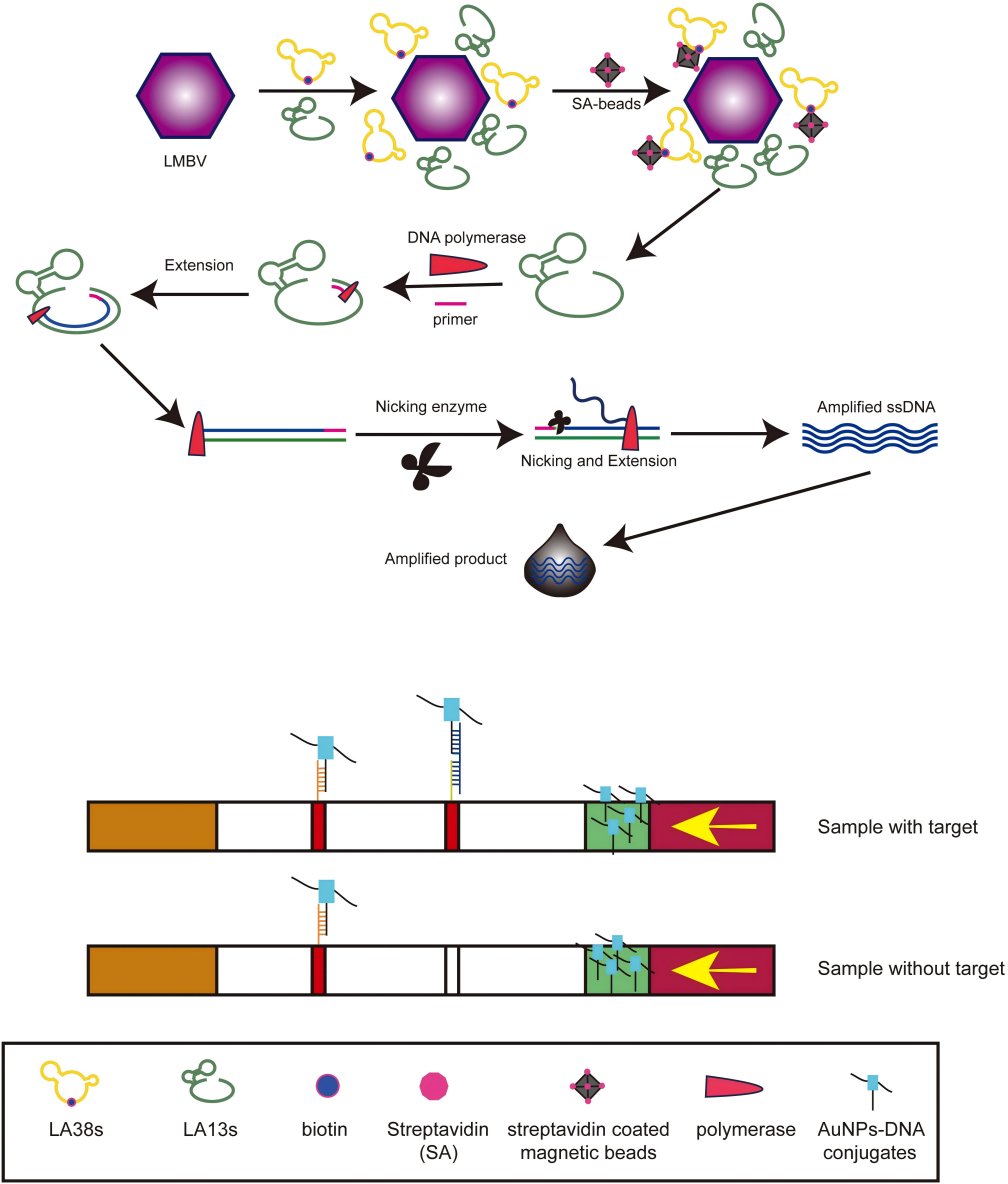
The procedure for LMBV detection by LFB. After incubation of LA38s and LA13s with LMBV, SA-modified magnetic beads were added, and the capture-aptamer-LMBV-amplification-aptamer-magnetic beads complex was collected through a magnetic separator. After SDA reaction, the product was added to the sample pad for detection.

The detection process of the LFB was illustrated in Figure 2. Firstly, the capture-aptamer (500 nM) and amplification-aptamer (500 nM) were incubated with LMBV in 100 μL PBS for 1 h. Subsequently, 1.5 μL of SA-modified magnetic beads were added, and the mixture was shaken at 4 °C for 30 min. The capture-aptamer-LMBV-amplification-aptamer-magnetic beads complex was collected by a magnetic separator. After washing three times of PBST (PBS supplied with 0.1% Tween-20), the complex was resuspended in 10 μL of ultrapure water and used as a template for the SDA reaction. As shown in Table 2, the SDA reaction system mainly consisted of primer, Nt.*Bbv*CI enzyme and Klenow Fragment exo-DNA polymerase, performed at 37 °C for 30 min. The amplification-aptamers were modified with Nt.*Bbv*CI recognition sites. The reaction starts with the hybridization of the primer and primer binding sequence, followed by DNA polymerase extension. The newly formed complementary chain was cleaved at the Nt.*Bbv*CI cleavage site, and then the extension reaction continued at the cleavage site. This process was repeated, and a large amount of ssDNA was amplified. Finally, the ssDNA were added to the sample pad of LFB. After rinsing with 30 μL of saline-sodium citrate (0.6 M NaCl, 0.06 M sodium citrate, pH = 7.0), the detection results of LFB can be observed within 5 min.

**TABLE 2:** Reaction system of SDA.

Materials	Volume
Nt. *Bbv*CI (10000 U/mL)	0.5 μL
Klenow fragment exo-DNA polymerase (5000 U/mL)	0.8 μL
Polymerase Klenow fragment exo- reaction buffer-2 (10×)	2 μL
Primer (10 μM)	2 μL
dNTPs (25 μM)	2 μL
Template	10 μL
ultrapure water	12.7 μL
Total volume	20 μL

### 2.8 The optimum working condition

Briefly, LMBV-infected FHM cells at a concentration of 8 × 10^7^/mL were used as the test samples, while uninfected FHM cells served as the control. In order to obtain the optimal temperature of the SDA reaction, we set the temperature gradient (25 °C, 37 °C, 42 °C, 50 °C, and 65 °C) for testing. In addition, in order to shorten the detection time, we first changed the incubation time of the sample, capture-aptamer and amplification-aptamer (2 min, 5 min, 10 min, 20 min, and 30 min). Subsequently, we varied the incubation time of the the capture-aptamer-sample-amplification-aptamer complex and magnetic beads (2 min, 5 min, 10 min, 20 min, and 30 min) to test the assay performance of the LFB.

### 2.9 Sensitivity analysis of LFB

Briefly, LMBV-infected FHM cells were diluted to various concentrations: 8 × 10^7^,8 × 10^6^, 8 × 10^5^, 8 × 10^4^, 8 × 10^3^, 8 × 10^2^ and 8 × 10^1^/mL. Uninfected FHM cells were used as the control group. The cells were collected, lysed and tested for the LFB as described above. In order to detect accurately, LFB was scanned by a portable test strip reader (Hangzhou Allsheng, Zhuantang Town, China). It can record the optical intensity of the C-line and the T-line.

### 2.10 Sample preparation for LFB and PCR detection

Thirty largemouth bass (10 ± 5 cm in length) with suspected LMBV infection were collected from farms in Guangdong Province, China. These fish were dissected and the spleen tissue were divided into two parts, one for LFB detection and the other part was used to extract DNA for PCR detection.

Sample preparation and testing procedures for LFB are as described above. DNA from spleen tissue was extracted using the TIANGEN DNA extraction kit. The PCR system was prepared using the LA Taq (TAKARA) kit as shown in Table 3. Primers (forward: ATTATCCCGTGGGTTGGTT and reverse: CGATGGGCTTGACTTCTCC) specific for the gene (ORF579) encoding the LMBV MCP protein were used for PCR detection. According to the reaction conditions shown in Table 4, 35 cycles were performed by the PCR instrument to amplify the product. Subsequently, 7 μL PCR product was diluted with 10-fold loading buffer (Takara, Shiga, Japan) and coated on 1% (w/v) agarose gel containing ethidium bromide. The agarose gel was electrophoresed in 1 × TAE buffer at 170 V constant voltage for 15 min. Finally, it was placed in a gel imager for imaging analysis.

**TABLE 3:** The PCR system.

Materials	Volume
DNA	1 μL
LA Taq	0.25 μL
10 × Buffer	2 μL
dNTP	2 μL
Primer F	1 μL
Primer R	1 μL
ddH_2_O	12.75 μL
Total Volume	20 μL

**TABLE 4:** The PCR reaction conditions.

Temperature	Time
95 °C	3 min
95 °C	30 s
60 °C	30 s
72 °C	1 min
72 °C	5 min

## 3 Results

### 3.1 Characterization of synthesized AuNPs- probe conjugates

**FIGURE 3:**
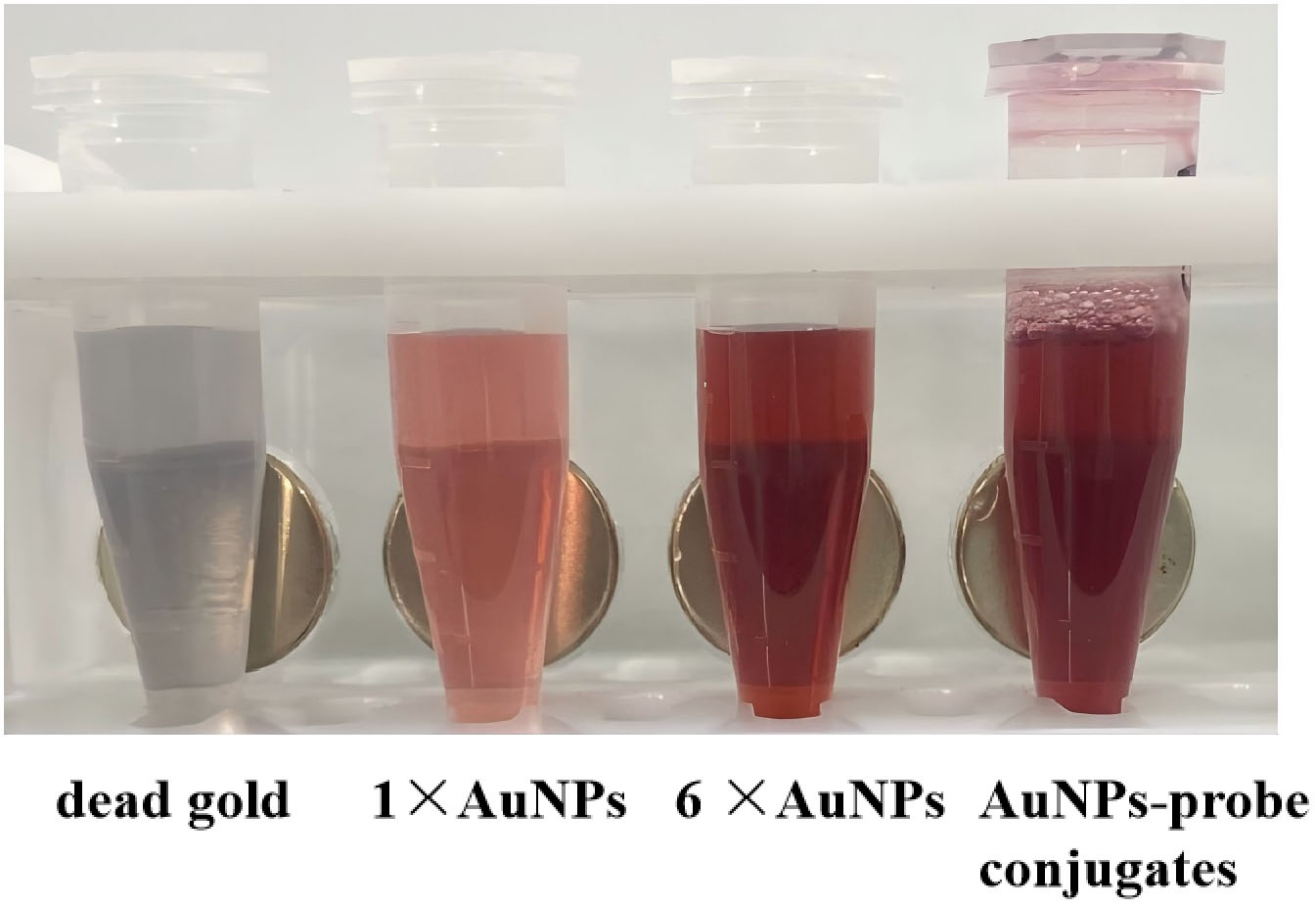
The preparation of AuNPs-probe conjugates. From left to right: dead gold caused by excessive NaCl, AuNPs solution, concentrated 6 times of AuNPs solution, AuNPs-probe conjugates solution.

As shown in Figure 3, the AuNPs solution had a transparent red with no precipitation. After 6 times of concentration, concentrated AuNPs turned to wine red. The 6 × AuNPs was coupled with the probe, blocked and aged to obtain AuNPs-probe conjugate. The conjugate was dark red, stable, clear and transparent.

### 3.2 Validation of the biosensor

**FIGURE 4:**
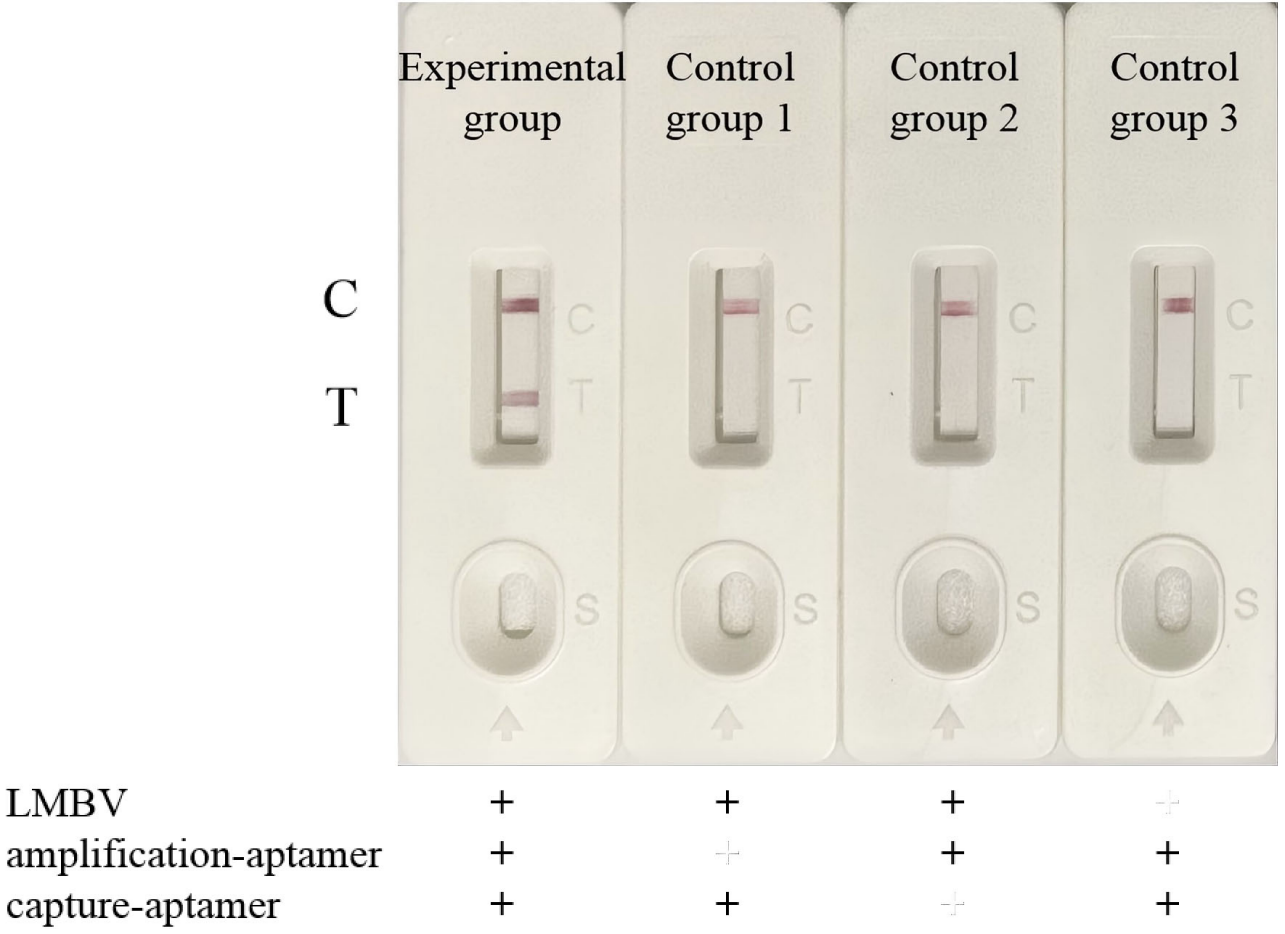
Verification of the LFB for LMBV detection. From left to right: Experimental group, LMBV was co-incubated with amplification-aptamer and capture-aptamer. Control group 1, LMBV was incubated with capture-aptamer. Control group 2, LMBV was incubated with the amplification-aptamer. Control group 3, PBS was incubated with amplification-aptamer and capture-aptamer. T-line could only be observed obviously in the presence of LMBV, amplification-aptamer and capture-aptamer. In the absence of LMBV, capture-aptamer or amplification-aptamer, LFB only showed C-line, and no positive T line appeared. The experiment was performed in triplicates.

After assembling the LFB, we designed four group of experiments to verify the biosensor for LMBV detection. In the experimental group, LMBV (MOI = 10) was incubated with both the amplification-aptamer (LA13s) and the capture-aptamer (LA38s). In control group 1, LMBV was incubated with only capture-aptamer. In control group 2, LMBV was incubated with only amplification-aptamer. In control group 3, PBS was incubated with both the amplification-aptamer and the capture-aptamer. The detection results were shown in Figure 4. Only when LMBV coexists with the amplification-aptamer and the capture-aptamer, the T-line and C-line of LFB can become red. In the three control groups, only the C-line showed red color, with no color change on the T-line.

The capture-aptamer was biotinylated, allowing it to non-covalently bind to SA-modified magnetic beads, thereby enriching the captured target in the magnetic beads. The amplification-aptamer has an enzyme cleavage site, which is a necessary condition for the SDA reaction. Only when the capture-aptamer, amplification-aptamer and target exist, the capture-aptamer-LMBV-amplification-aptamer-magnetic bead complex can be formed, and ssDNA can be amplified by SDA reaction. The ssDNA, which is complementary to the sequence of T-line probe and the sequence of AuNPs-probe, produced a red strip in the T-line region. Additionally, the sequence of AuNPs-probe was also complementary to the sequence of C-line probe. Under the action of capillary force, the excessive AuNPs-probe conjugate complex continued to migrate, and color reaction occured in the C-line region. When there is no LMBV in the detection system, the complex cannot be formed. In the absence of capture-aptamers or amplification-aptamers, the target cannot be enriched by magnetic beads or SDA reaction cannot be performed. Therefore, only when the LMBV, capture-aptamer and amplification-aptamer are present, the positive T line can be seen. In the absence of target, capture-aptamer or amplification-aptamer, the positive T-line could not be observed. The above results showed that the LFB was able to effectively detect LMBV.

### 3.3 Specificity of the LFB

**FIGURE 5:**
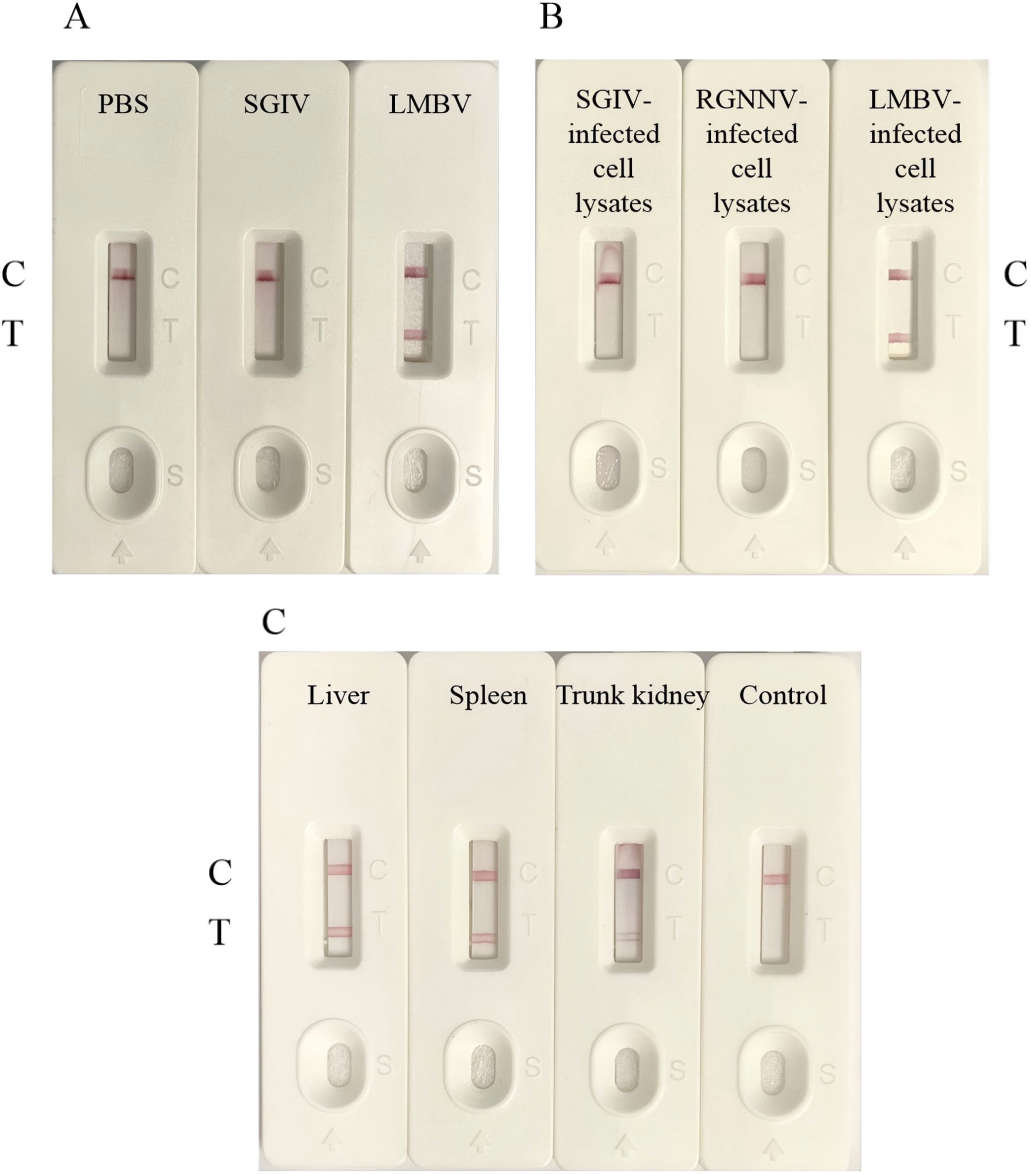
The specificity of LFB. **(A)** LFB could specifically detect LMBV particles. **(B)** LFB could specifically detect LMBV infected cell lysates. **(C)** LFB could specifically detect the liver, spleen and kidney tissues of largemouth bass infected with LMBV. The experiment was performed in triplicates.

To evaluate the specificity of LFB, different purified viruses, virus-infected cell and virus-infected tissue were used as samples to be tested. The PBS or uninfected tissue were used as the control groups. As shown in Figure 5A, LMBV particle samples showed a clear and visible T-line, while SGIV particle samples and control group only showed C-line, without positive T-line. As shown in Figure 5B, only LMBV-infected FHM cell samples were detected positive, while SGIV-infected GS cell samples and RGNNV-infected GB cells samples did not show positive T lines. Furthermore, biosensors were used to detect the liver, spleen and body kidney tissue samples of the infected largemouth bass. As shown in Figure 5C, the three tissue samples all displayed the visible T-line. These results demonstrated that the LFB had high specificity for LMBV detection, which can effectively detect LMBV particles, LMBV-infected cells and tissue samples.

### 3.4 Optimization of experimental conditions

**FIGURE 6:**
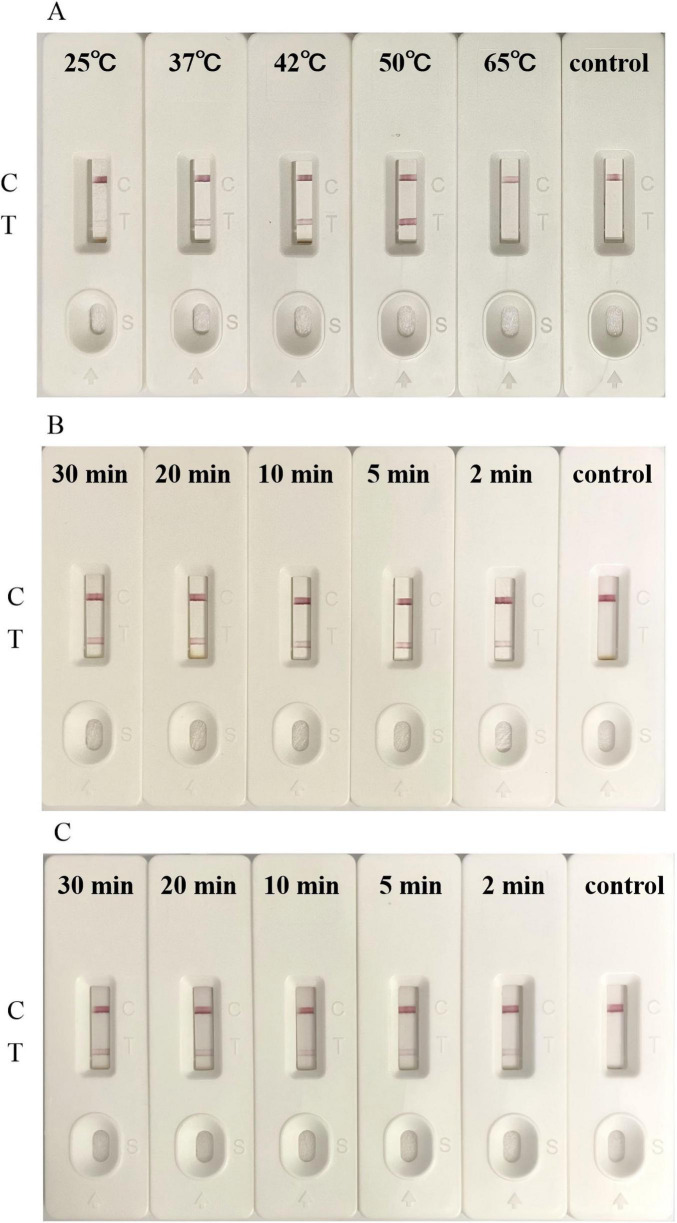
Optimization of LFB for detection. **(A)** The optimal SDA temperature for the detection of LMBV infection by LFB was 50 °C. **(B)** The LFB detected LMBV infection with an incubation time for sample and two aptamers as short as 2 min. **(C)** The optimal incubation time between the capture-aptamer-sample-amplification-aptamer complex and magnetic beads was 20 min. Uninfected FHM cells were used as the control. The experiment was performed in triplicates.

In order to further improve the detection efficiency of LFB, we optimized some detection steps. We collected and lysed 8 × 10^7^ LMBV-infected FHM cells for LFB detection. Uninfected FHM cells were used as the control. Firstly, different temperatures (25 °C, 37 °C, 42 °C, 50 °C, and 65 °C) were set to be applied to SDA. When the reaction temperature was as low as 25 °C or as high as 65 °C, the SDA reaction could not proceed properly, and the LFB displayed only a C-line without T-line (Figure 6A). When the reaction temperature was 37 °C, 42 °C and 50 °C, the SDA reaction can proceed normally, and the LFB showed a clear red detection line. It is worth noting that the T-line band of the 50 °C experimental group was significantly darker than the other two groups (Figure 6A). This indicated that the SDA reaction has higher amplification efficiency under this condition, so that the biosensor could capture more targets. Subsequently, we reduced the incubation time of the sample and two aptamers to determine the effect of incubation time. Even though the incubation time was reduced to 2 min, the LFB could still showed a light red T-line (Figure 6B). In addition, we also changed the incubation time of capture-aptamer-sample-amplification-aptamer complex and magnetic beads. As shown in Figure 6C, when the incubation time was 2 min, 5 min and 10 min, LFB all showed a red T-line, but the color of the T-line became darker with the increase of incubation time. We believed that this phenomenon was due to the fact that the binding reaction between the magnetic beads and the capture-aptamer had not yet reached the upper limit. There was almost no difference in the color of the detection line between the 20 min experimental group and the 30 min experimental group. Therefore, the experimental results showed that 20 min could fully combine the magnetic beads with the capture-aptamer-sample-amplification-aptamer complex. The above results showed that the optimal reaction temperature of SDA was 50 °C. After optimizing the LFB detection procedure, the whole detection process could be completed within 1 h.

### 3.5 Sensitivity of the LFB

**FIGURE 7:**
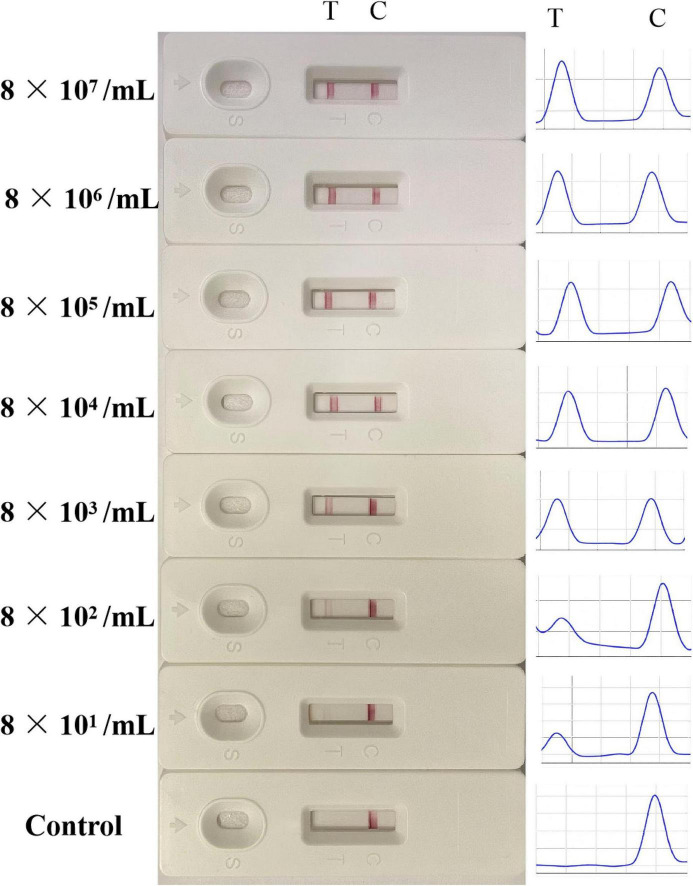
The sensitivity analysis of the LFB. Various concentrations of LMBV-infected FHM cells (8 × 10^7^, 8 × 10^6^, 8 × 10^5^, 8 × 10^4^, 8 × 10^3^, 8 × 10^2^ and 8 × 10^1^/mL) were prepared, and uninfected FHM cells were used as control group. The experiment was performed in triplicates.

The sensitivity of LFB was verified by various concentrations of LMBV-infected FHM cells. As shown in Figure 7, in the control group, no positive T-line was observed. When LMBV-infected FHM cells were as low as 8 × 10^1^/mL, their lysates could still be detected by LFB. At the same time, the peaks corresponding to the T-line and the C-line were observed by the strip reader. The peak area of the T-line decreased with the decrease of the concentration of LMBV-infected cells. Thus, these results suggested that the detection limit of the LFB was 8 × 10^1^/mL.

### 3.6 Sample for LFB and PCR detection

**FIGURE 8:**
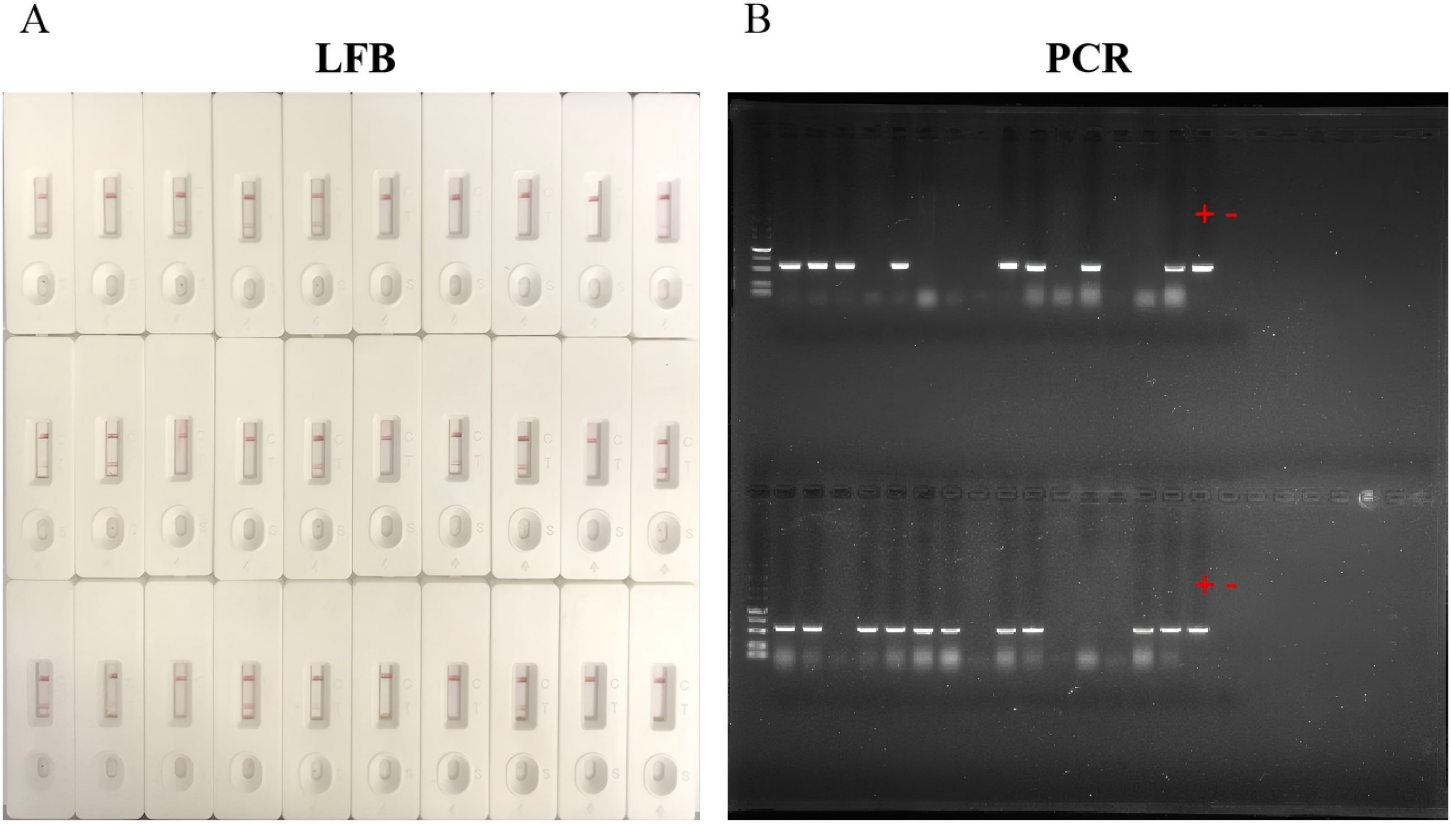
Detection of largemouth bass. **(A)** 14 positive samples were detected by LFB. **(B)** 18 positive samples were detected by PCR. “+” represents the positive control sample, “–” represents the negative control sample.

The existence of LMBV in 30 largemouth bass collected from farms was detected by both LFB and PCR. The results of LFB showed that 14 (46.7%) samples were positive of LMBV and 16 (60%) samples were negative of LMBV (Figure 8A). Among them, 18 were confirmed positive by the PCR (Figure 8B). This means that 4 (13.3%) samples were positive by PCR, but LFB were negative (Table 5). Overall, the accuracy of LFB was slightly lower than that of PCR, but LFB was able to obtain the results within 1 h.

**TABLE 5:** Comparison of positive detection rate between LFB and PCR.

Method	Tested samples	Positive samples	Positive ratios
LFB	30	14	46.7%
PCR	30	18	60%

## 4 Discussion

The frequent outbreaks of disease caused by LMBV have severely interfered the healthy development of the largemouth bass industry. The clinical symptoms of LMBV infection are variable and complicated, making it difficult to diagnose by symptom signs (Jin et al., 2022). This poses new challenges for detection. The results of clinical symptoms and pathological observations can only be used as auxiliary judgments. Accurate diagnose is essentially important for preventing and controlling disease. Hence, rapid and on-site detection methods for LMBV are necessary.

For the detection of LMBV, multiple nucleic acid amplification techniques have been developed, such as droplet digital PCR (ddPCR), qRT-PCR, LAMP, and RPA. The lowest detection limit of RT-qPCR for LMBV was 5.8 copies/μL, with a 100% positive detection rate (Guo et al., 2022). Jiang et al. (2023) developed a ddPCR method that detect as low as 2 copies/μL. Guang et al. (2024) employed recombinase-aided amplification technology coupled with CRISPR and Cas13a for LMBV detection, achieving a detection limit as low as 3.1 × 10^1^ copies/μL. Similarly, a detection method based on RPA-CRISPR/Cas12 was established, achieving a sensitivity of 50 copies/reaction within 40 min (Li et al., 2025). The LAMP assay targeting the LMBV MCP gene is highly specific for LMBV diagnosis, as it shows no cross-reactivity with large yellow croaker iridovirus or SGIV (Zhu et al., 2020). Additionally, an RPA assay targeting the LMBV MCP gene was developed for LMBV detection, with a detection limit of 89 copies/μL (Zhu et al., 2022). In this study, the LFB demonstrated high detection specificity for LMBV without cross-reactivity with RGNNV or SGIV. This method achieved a lower detection limit of 8 × 10^1^/mL LMBV-infected cell. However, LFB does not target specific viral genes and eliminates the need for nucleic acid extraction steps.

To date, various aptamer-based LFBs have been reported, which are increasingly recognized as user-friendly detection tools. They have been applied in areas such as pathogens, hormones, and disease-associated biomarkers. Based on aptamer (Np-A48), an ultrasensitive electrochemical aptamer-based aptasensor for SARS-CoV-2 detection was developed using CRISPR/Cas12a, achieving a detection limit of 16.5 pg/mL (Han et al., 2022). Xu et al. (2025) developed an ultrasensitive surface-enhanced Raman spectroscopy technique using aptamer-antibody dual-recognition elements for rapid detection of intraoperative parathyroid hormone. There is also an LFB that utilizes aptamers to capture targets for the detection of human osteopontin protein (OPN), achieving qualitative and semi-quantitative detection of OPN in the serum of cancer patients at clinical cutoff levels (Mukama et al., 2020).

In field testing, LFBs demonstrated a detection rate of 46.7%, slightly lower than PCR. Nevertheless, they maintain significant advantages in operational simplicity and rapid detection. Furthermore, sensitivity of LFBs can be further improved through optimization of both aptamers and the detection system. Aptamers are the key recognition element of the LFB method, so their properties largely determine the detection sensitivity. Aptamers can be further optimized in different ways such as truncation, chemical modification, mutation of nucleic acid base, and construction of multivalent aptamers. For example, the truncation of aptamers against Gonyautoxin 1/4 obviously increase the binding affinity, with a higher Kd of 17.7 nM (Gao et al., 2016). The aptamers used in this study were also truncate, and showed higher affinity compared with the original aptamers.

Several rapid detection methods have been developed based on aptamer-designed detection systems combined with SDA. For example, an aptamer-mediated double SDA-microchip electrophoresis method achieved a detection limit as low as 6 CFU/mL for *Salmonella Typhimurium* (Lu et al., 2024). Lu et al. (2025) developed an A-LKS-mediated SDA-Cas12a signal cascade biosensor for ultrasensitive aflatoxin B1 detection. Islam et al. (2021) developed a label-free method for detecting miR-215 in saliva and serum using aptamers, thioflavin T dye, and SDA. In this study, LA38s and LA13s with high affinity and specificity of LMBV particles were used to capture target and initiate subsequent SDA reaction, respectively. SDA reaction could be conducted under isothermal condition, and reaction product further amplified detection signal (Zhang et al., 2021). Combined with aptamers and SDA, a novel LFB was developed for visual detecting of LMBV, without requiring special equipment and laboratory conditions. After optimization, the detection for LMBV could be completed in 1 h, with detection limit of 8 × 10^1^/mL LMBV infected cells. These two aptamers had been generated and applied in developing aptamer-based sandwich enzyme-linked apta-sorbent assay (ELASA) for LMBV detection. The detection limit of ELASA was 1.25 × 10^2^/mL LMBV-infected cells, and the detection time required approximately 4 h (Zhang et al., 2022b). Obviously, the LFB showed higher sensitivity and shorter detection time, compared with that of ELASA.

Currently, the development of aptamer-based LFB for aquatic animal virus detection remains at an early stage. Building upon the RGNNV-specific aptamer, Liu et al. (2020) developed a sensitive LFB capable of detecting RGNNV-CP protein at concentrations as low as 5 ng/mL. In another study, researchers developed an LFB for rapid SGIV detection by employing two SGIV-specific aptamers, achieving detection within 90 min (Liu et al., 2021). Several studies have integrated nucleic acid amplification techniques with LFB to achieve visual detection (Preena et al., 2022). For instance, rapid detection LFBs have been developed targeting specific viral genes, such as the SS N gene of *Micropterus salmoides* rhabdovirus (MSRV), as well as the MCP and ORF 007 genes of infectious spleen and kidney necrosis virus (ISKNV; Feng et al., 2022; Li et al., 2020). In this study, two LMBV-specific aptamers were combined with SDA, and LFB was utilized to achieve visual detection. The detection limit of LFB is as low as 8 × 10^1^/mL LMBV infected cells, and the color change of the LFB can be observed by the naked eye within 5 min.

## 5 Conclusion

In conclusion, we developed a novel LFB based on specific aptamers, with high sensitivity, specificity and stability. It was time-saving, easy to handle, visualization of results, and did not need special equipment, thus providing a valuable option for rapid detection for LMBV in field. Thus, LFBs contribute to sustainable aquaculture by ensuring fish health, optimizing therapeutic interventions, mitigating disease-related financial risks, and enhancing production yields.

## Data Availability

The raw data supporting the conclusions of this article will be made available by the authors, without undue reservation.
